# Hybrid Effect Evaluation of Steel Fiber and Carbon Fiber on the Performance of the Fiber Reinforced Concrete

**DOI:** 10.3390/ma9080704

**Published:** 2016-08-18

**Authors:** Weimin Song, Jian Yin

**Affiliations:** 1 Department of Civil and Environmental Engineering, The University of Tennessee, Knoxville, TN 37996, USA; 2 School of Civil and Mechanics Engineering, Central South University of Forestry and Technology, Changsha 410004, China; T20111776@csuft.edu.cn

**Keywords:** steel fiber, carbon fiber, hybrid fiber reinforced concrete, compressive toughness, impact toughness, hybrid effect

## Abstract

Fiber reinforcement is an important method to enhance the performance of concrete. In this study, the compressive test and impact test were conducted, and then the hybrid effect between steel fiber (SF) and carbon fiber (CF) was evaluated by employing the hybrid effect index. Compressive toughness and impact toughness of steel fiber reinforced concrete (SFRC), carbon fiber reinforced concrete (CFRC) and hybrid fiber reinforced concrete (HFRC) were explored at steel fiber volume fraction 0.5%, 1%, 1.5% and carbon fiber 0.1%, 0.2%, 0.3%. Results showed that the addition of steel fiber and carbon fiber can increase the compressive strength. SF, CF and the hybridization between them could increase the compressive toughness significantly. The impact test results showed that as the volume of fiber increased, the impact number of the first visible crack and the ultimate failure also increased. The improvement of toughness mainly lay in improving the crack resistance after the first crack. Based on the test results, the positive hybrid effect of steel fiber and carbon fiber existed in hybrid fiber reinforced concrete. The relationship between the compressive toughness and impact toughness was also explored.

## 1. Introduction

Concrete is one of the most widely used construction materials, but the tensile strength is relatively low in contrast with the compressive strength. Generally, the ratio between uniaxial tensile strength and compressive strength is in the range 0.07 to 0.11 [1], this defect restricts the application of concrete in many aspects. With the development of the industry, many approaches to enhance the concrete’s performance came into existence. As one of the commonly used methods, fiber reinforcement is becoming more and more widely considered nowadays [1,2,3].

Steel fiber and carbon fiber are the two most widely used fibers in the concrete industry. Lots of pioneering work has been done on the workability, strength and durability of the steel fiber reinforced concrete (SFRC) and carbon fiber reinforced concrete (CFRC). Generally speaking, the incorporation of fiber can decrease the workability of concrete [4,5]. Due to the high stiffness of steel fiber, the fiber shape does not change in the mix and vibration process. However, for polypropylene fiber or carbon fiber, the shape changes during the mix and vibration process because of the interaction between fiber and mortar or concrete, which can lead to worse fiber dispersion of carbon fiber in concrete in contrast with steel fiber. The length to diameter ratio and the fiber volume were two factoring affecting the workability of the fresh fiber reinforced concrete [6,7,8]. To better understand the fiber dispersion in concrete, fiber orientation-factor (0≤α≤1) was introduced [9,10,11]. This factor can be evaluated from the fiber volume ratio ($V_{f}$), the cross-section area of concrete specimen ($A_{c}$), number of fibers ($N_{f}$) in the cross-section area and the cross-section area of fiber ($A_{f}$), as shown in Equation (1). The larger the value of *α*, the better the fiber dispersion.

(1) $$\begin{array}{c} \alpha =\left(N_{f}A_{f}\right)/\left(V_{f}A_{c}\right) \end{array}$$

Regarding the mechanical property, research showed that by adding fiber within a certain fiber volume limit, the compressive strength and flexural strength could increase [1,12,13,14]. Some research also indicated that in the flexural test, fiber reinforced concrete showed hardening post-peak behavior, which played an important role in the flexural behavior [12,15]. Besides, the splitting tensile strength and modulus of the steel fiber-reinforced concrete increased as the volume of steel fiber increased [16]. By considering both the load and deformation, some studies introduced the compressive toughness to evaluate the effect of fiber on the concrete behavior and defined the compressive toughness as the area under the compressive stress-strain curve [17,18], which is the energy density of the concrete under the compression test. Research showed that the compression toughness can be significantly increased when fiber was incorporated [16]. Hsu, L.S.M., Hsu, C.T.T. [19] and Taerwe, L.R. [20] proposed the concept of toughness index (TI), which is the compression toughness ratio between fiber reinforced concrete and natural concrete without fiber added. The strain corresponding to the peak stress under compression test can also increase as the addition of fiber, and as the volume increases, the strain increases as well [21,22,23]. Nataraja et al. [22] made an analysis of the stress-strain relation of SFRC under compression, evaluated the toughness and set up the stress-strain equation and made a comparison with plain concrete. The constitutive behavior of concrete confined with fiber [24,25] was also explored by many researchers to evaluate the effect of the external constraint. Besides, the impact toughness and fatigue property also increase considerably [1,26] when the fiber was added, both the numerical and experimental analysis results indicated that increasing the fiber volume fraction increased the impact resistance of the concrete specimens and steel fiber showed better performance than polypropylene fiber in improving the impact resistance [26].

Because of the well mixed design and selection of fibers, fiber hybridization is an effective approach to enhance the performance of concrete compared to single type or single size fiber [4,13,14,27,28,29]. Fiber hybridization often falls into two categories. One hybridization is achieved by different types of fibers, such as steel-carbon fiber, steel-polypropylene fiber, etc. [13,29,30,31]. This kind of hybridization often enhances the concrete performance based on fiber constitutive response [4], which means one type of fiber is stronger and stiffer, the other one is relatively flexible, these two types of fibers play different roles in the different period of the concrete failure. Hybridization of fiber with different dimensions belongs to another category, the enhancement is achieved by the reinforcement from micro-scale to macro-scale [4,27].

## 2. Objective

The objective of this study was to investigate the hybrid effect of steel fiber and carbon fiber by the evaluation of the compression and impact properties. The compressive toughness and impact toughness of concrete with different fiber fraction were explored. The hybrid parameter was also obtained to characterize the hybridization effect.

## 3. Experimental Program

### 3.1. Materials

**Figure 1:**
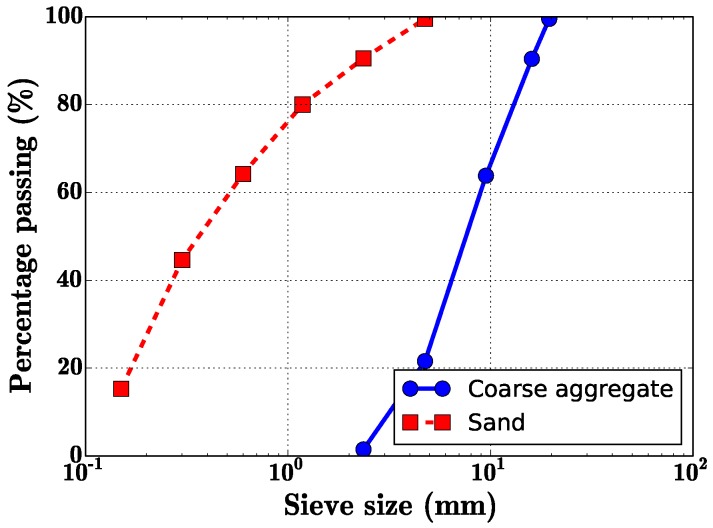
Aggregate gradation.

**Figure 2:**
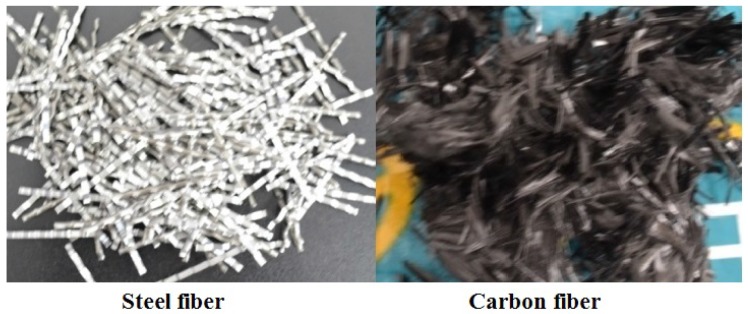
Steel fiber and carbon fiber.

The materials for the concrete in the paper are as follows:

- Cement: P.O 42.5 cement was selected, P.O 42.5 means ordinary portland cement and the 28-day cement mortar strength is 42.5 MPa when the water-cement ratio is 0.45. The chemical components of the cement is shown in Table 1.
- Aggregate: gravel aggregate with the nominal maximum aggregate size (NMAS) 19.5 mm.
- Sand: rive sand with the fineness modulus 2.78, the aggregate gradation of the coarse aggregate and sand can be seen in Figure 1.
- The chemical components of the fly ash is shown in Table 1.
- Water reducer: polycarboxylic high efficiency water reducer.
- Fiber: steeel fiber and carbon fiber were shown in Figure 2, properties of the two fibers can be seen in Table 2.

### 3.2. Mixing and Curing

In efforts to ensure the properly fiber dispersion in composite, a two-stage mixing procedure was performed. The fiber, cement, fly ash and aggregate were mixed first in one mechanical mixer for 3 min and then the water and superplasticizer were added in the composite to mix for another 3 min. The specimen size for compression test is 100 mm × 100 mm × 300 mm, ϕ150 mm × 60 mm for the impact test.

After mixing, a vibrating table was used to ensure the good compaction. The surface of the specimen was then smoothed, and waterproof membrane was used to cover the surface of the concrete until the specimens were demolded 24 h after being casted. The specimens were cured at an air temperature 20 ± 2 ${}^{\circ }$C and at a relative humidity of 95%. After 28 days curing, the compression test and impact test were conducted.

### 3.3. Concrete Mix Design

The concrete mix proportions of all mix design were shown in Table 3. The water cement ratio is 0.46. The fly ash and the superplasticizer were added in the concrete to ensure the good workability. The volume fraction of steel fiber is 0.5%, 1.0% and 1.5%, the volume fraction of carbon fiber is 0.1%, 0.2% and 0.3%.

### 3.4. Uniaxial Compression Test

**Figure 3:**
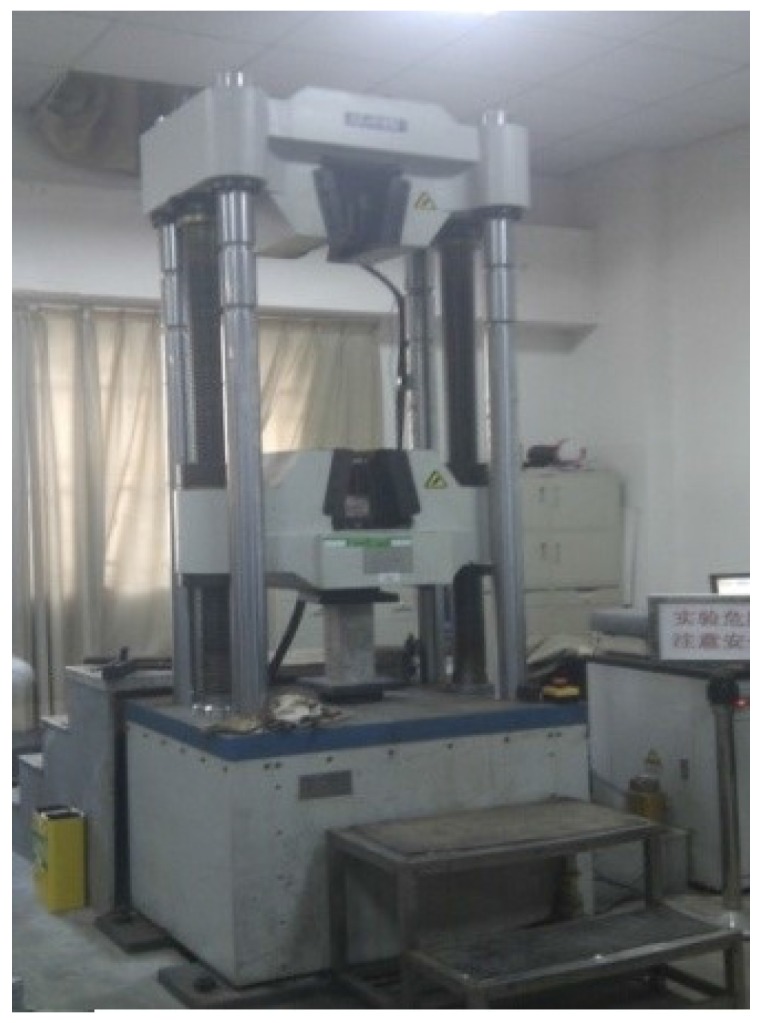
Hydraulic servo testing machine.

The uniaxial compression test was conducted using hydraulic servo testing machine (SANS, Shenzhen, China), the applied load and deformation of the concrete were recorded simultaneously. The machine is shown in Figure 3. The uniaxial compression test was conducted in triplicate.

**Figure 4:**
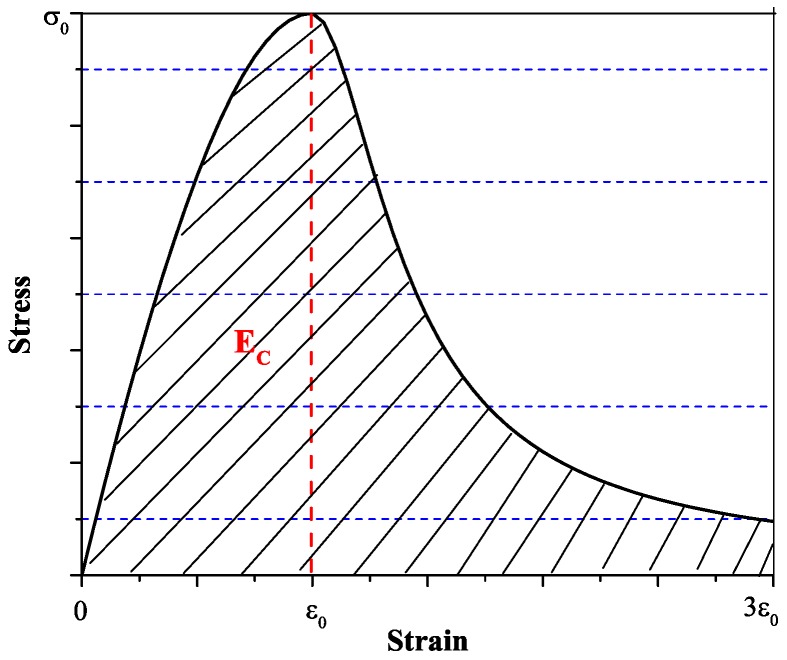
Stress-strain curve.

The compressive toughness was selected in this part to analyze the compressive behavior. Some studies regard the compression toughness as the area under the stress-strain curve [17,18], which is the energy density of the concrete under the compression test. In this study, to make a comparison with impact toughness (see Section 4.2), compression toughness is defined as the area under the load-deformation curve. In this study, the compression toughness can be obtained from Equation (2), *σ* is the compressive strength, $\epsilon_{0}$ is the strain corresponding to the peak strength ($\sigma_{0}$), the strain interval for toughness calculation is $0∼3\epsilon_{0}$, as shown in Figure 4.

(2) $$\begin{array}{c} E_{C}=V\int_{0}^{3\epsilon_{0}}\sigma \cdot d\epsilon \end{array}$$

where *V* is the volume of the concrete, which is 0.003 m${}^{3}$.

### 3.5. Impact Test

**Figure 5:**
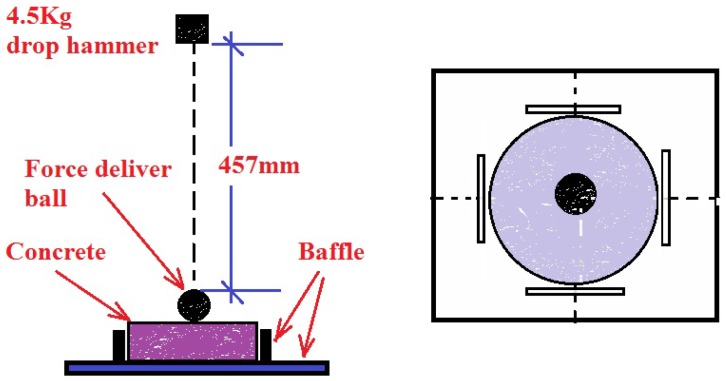
Impact test.

Based on ACI-544 [32], the drop-weight test was conducted to evaluate the impact toughness of fiber reinforced concrete, as shown in Figure 5. The impact resistance can be evaluated by the blow number of the first crack occurs and the number when the ultimate failure occurs. A 4.5 kg drop hammer was dropped from a height of 457 mm, the impact force was delivered from the 64 mm diameter ball to the concrete specimen. The hammer was dropped repeatedly, and the number of blows when the first visible crack happened on the top surface of the specimen and the ultimate failure happened were both recorded. Ultimate failure is the failure state that the pieces of concrete are touching three or four positioning lugs on the baseplate [32]. The impact test was conducted in triplicate.

In this study, the impact toughness was defined as the energy absorbed by the concrete transformed from the potential energy of the drop hammer during the failure process. The calculation of the impact toughness was followed.

(3) $$\begin{array}{c} E_{I}=N\cdot mgh \end{array}$$

where *N* is the impact number, the number of first visible crack and the final failure were respectively used, *m* is the mass of hammer, *h* is the falling height of the hammer, the values of *m* and *h* can be read from Figure 5.

## 4. Results

To evaluate the hybrid effect of the compression toughness and the impact toughness, hybrid effect index $\alpha_{R}$ was employed in this study [33].

(4) $$\begin{array}{c} \alpha_{R}=\frac{R_{H}-R_{0}}{\left(R_{i}-R_{0}\right)\beta_{i}} \end{array}$$

(5) $$\begin{array}{c} \beta_{i}=\frac{V_{i}}{V} \end{array}$$

(6) $$\begin{array}{c} \Sigma \beta_{i}=1 \end{array}$$

where $\alpha_{R}$-hybrid effect index; $R_{H}$-compression toughness or impact toughness of HFRC; $R_{0}$-compression toughness or impact toughness of natural concrete (NC); $R_{i}$-compression toughness or impact toughness of concrete incorporated with single SF or CF; $\beta_{i}$-the volume fraction of SF or CF in the whole volume of SF and CF; $V_{i}$-volume of SF or CF; *V*-volume of the whole fibers.

It should be noted that in the calculation of $R_{i}$, the fiber volume should be the same as the volume of the hybrid fiber used for $R_{H}$, by data fitting of the existing data of compressive toughness or impact toughness, $R_{i}$ can be obtained at different fiber volume fraction.

So, when $\alpha_{R}>1$, the hybrid effect is positive; when $\alpha_{R}<1$, the hybrid effect is negative.

### 4.1. Compression Test

**Figure 6:**
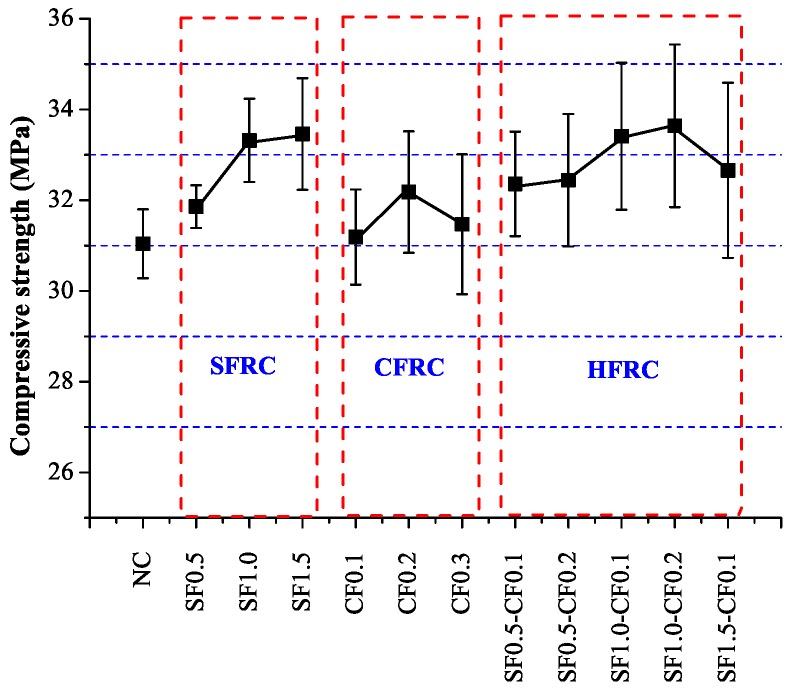
Compressive strength.

Figure 6 represents the compressive strength of the test. It can be clearly observed that as the steel fiber volume increased, the compressive strength of SFRC also increased. For CFRC with carbon volume 0.2% (CF0.2), the compressive strength was larger than normal concrete as well. The compressive strength of CF0.3 and SF1.5-CF0.1 were lower than that of CF0.2 and SF1.0-CF0.2, which may be caused by the non-uniform distribution of fibers when fiber volume increased to some extent.

**Figure 7:**
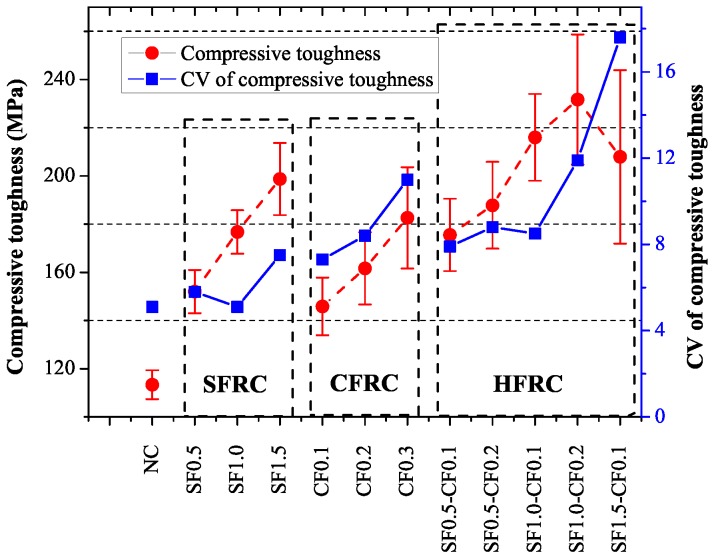
Compressive toughness.

Figure 7 shows the compression toughness of these specimens and the coefficient of variation (CV) of compression toughness. It can be observed apparently that for SFRC and CFRC, as the volume of SF or CF increased, the compressive toughness increased as well. For HFRC, the compressive toughness also increased with the increase of the fiber volume, and SF1.0-CF0.2 got the maximum compressive toughness value. The compressive toughness of SF1.5, CF0.3 and SF1.0-CF0.2 showed an increase of 75%, 63% and 95% respectively in contrast with NC. The compressive toughness of SF1.5-CF0.1 showed an opposite develop trend, which may be caused by the poor dispersion as the fiber volume increased. The coefficient of variation (CV) of compression toughness was shown in Figure 6, it can be apparently observed that as the volume of fiber increased, the coefficient of variation also increased, indicating that as the fiber volume increased, the scattering of the compressive toughness increased as well. The large coefficients of variation (CV) may be caused by the bad dispersion of the fibers as the fiber volume increased.

**Figure 8:**
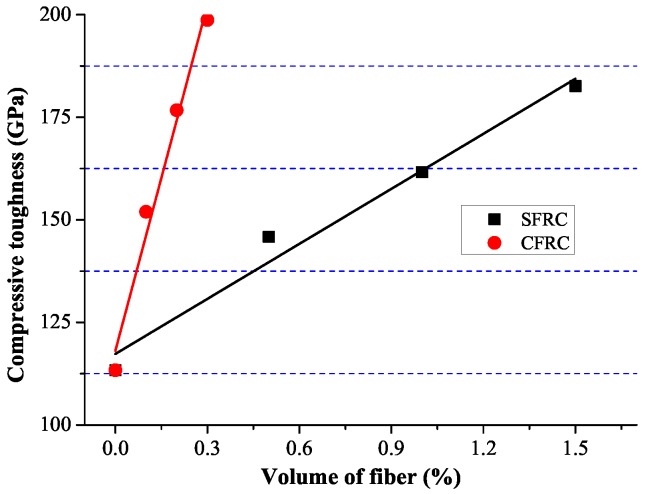
Linear regression between volume fiber and compression toughness.

For SFRC and CFRC, Figure 8 shows the relation between fiber volume and compression toughness. Linear correlation equations between fiber volume and compression toughness are shown as Equations (7) and (8).

(7) $$\begin{array}{c} \text{SFRC:}\;E_{C-S}=44.7V_{SF}+117.3\;\left(R^{2}=0.967\right) \end{array}$$

(8) $$\begin{array}{c} \text{CFRC:}\;E_{C-C}=280.9V_{CF}+118.1\;\left(R^{2}=0.972\right) \end{array}$$

where $E_{C-S}$ is the compressive toughness of SFRC, $E_{C-C}$ is the compressive toughness of CFRC.

**Figure 9:**
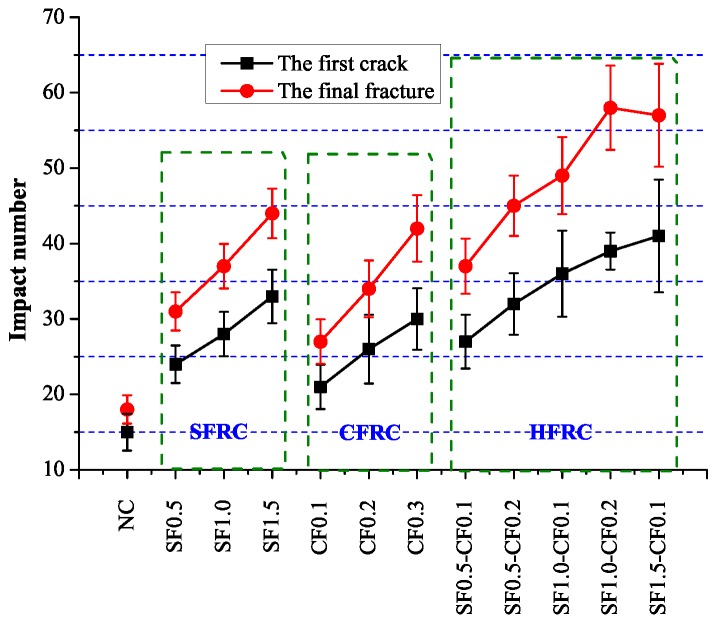
Hybrid effect index of compression toughness.

By using Equations (4)–(6), hybrid effect of HFRC can be calculated. Figure 9 shows the hybrid effect index of the compressive test. It can be seen that the hybrid effect index of all group of HFRC is larger than 1, indicating the compression toughness was not the simply sum of SFRC and CFRC, the hybridization of SF and CF played a positive effect on the compression toughness of HFRC.

### 4.2. Impact Test

**Figure 10:**
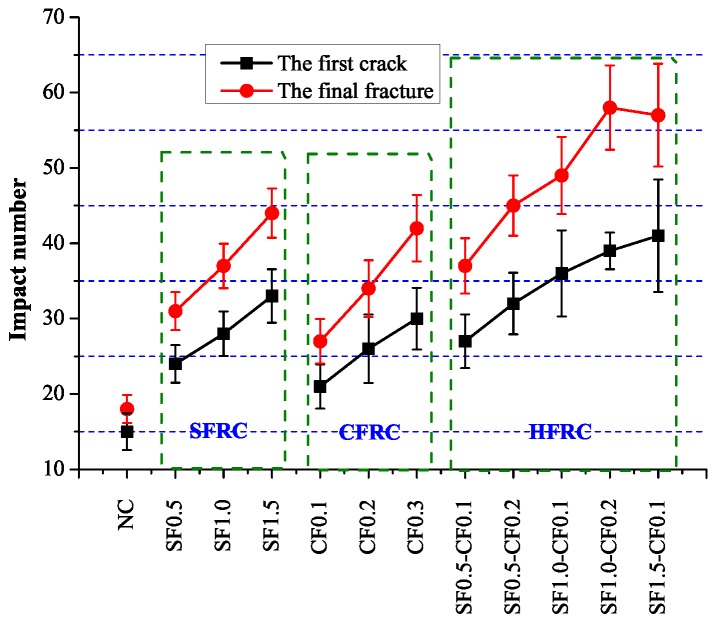
Test result of impact test.

**Figure 11:**
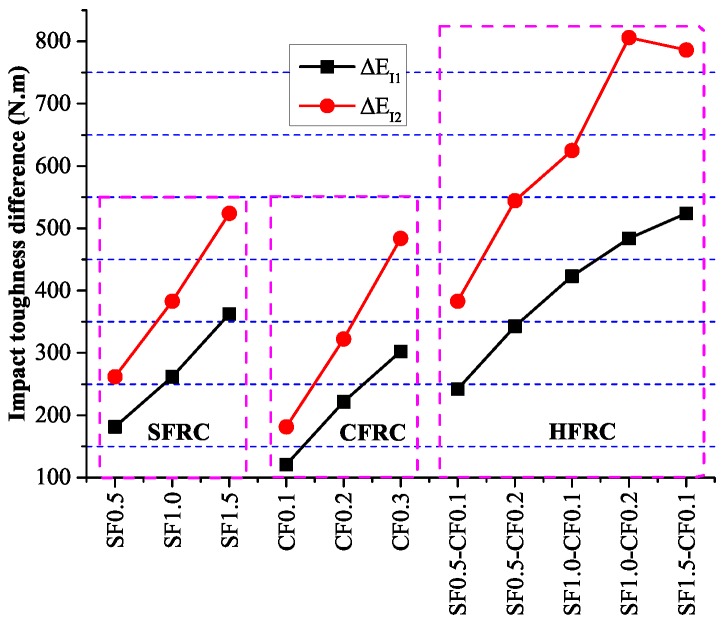
Impact toughness difference.

Impact test results were shown in Figure 10. It can be concluded that as the fiber volume increased, the impact number for the first crack and final fracture both increased considerably. The impact number for the final fracture of SF1.5, CF0.3 and SF1.0-CF0.2 were 44, 42 and 58 respectively, which increased 144%, 133% and 222% in contrast with NC. The impact number when the first crack occurred was 33, 30 and 41 for SF1.5, CF0.3 and SF1.0-CF0.2, which increased 120%, 100% and 173% respectively in contrast with NC. Figure 11 shows the impact toughness difference between fiber reinforced concrete and plain concrete, $\Delta E_{I1}$ defines the impact toughness difference when the first visible crack occurs and $\Delta E_{I2}$ is the impact toughness difference when the ultimate failure happens. It can be apparently obtained from Figure 11 that the impact toughness improvement after the first crack is larger than the improvement before the first crack, indicating fiber enhancement in the impact toughness mainly lies in the process after the first visible crack.

**Figure 12:**
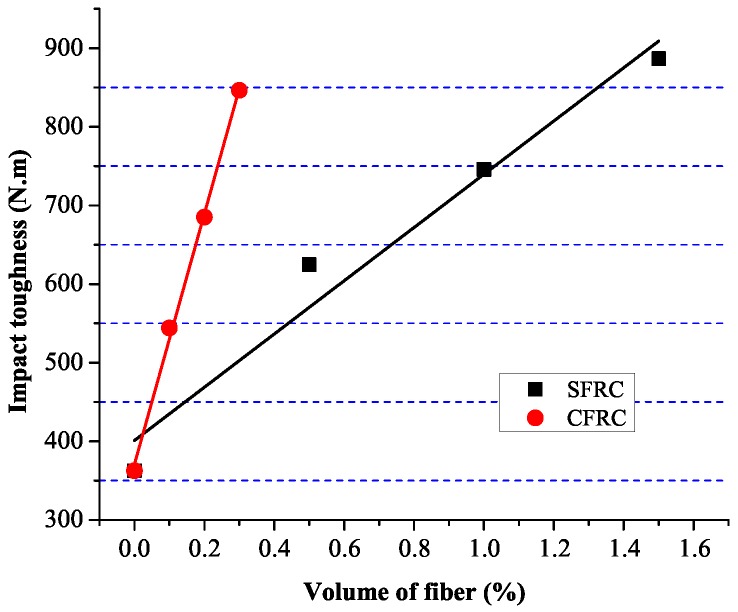
Linear regression bewteen volumes and impact toughness.

The energy absorption during the fracture of NC is used for the crack generation, development and penetration, but for fiber reinforced concrete, the energy is also used for the de-bonding between fiber and concrete. Figure 12 shows the relation between fiber volume and the impact toughness. Fitting equations were established between fiber volume and the impact toughness, as shown Equations (9) and (10).

(9) $$\begin{array}{c} \text{SFRC:}\;E_{I-S}=338.6V_{SF}+401.1\;\left(R^{2}=0.950\right) \end{array}$$

(10) $$\begin{array}{c} \text{CFRC:}\;E_{I-C}=1592.1V_{CF}+370.8\;\left(R^{2}=0.997\right) \end{array}$$

where $E_{I-S}$ is the impact toughness of SFRC, $E_{I-C}$ is the impact toughness of CFRC.

**Figure 13:**
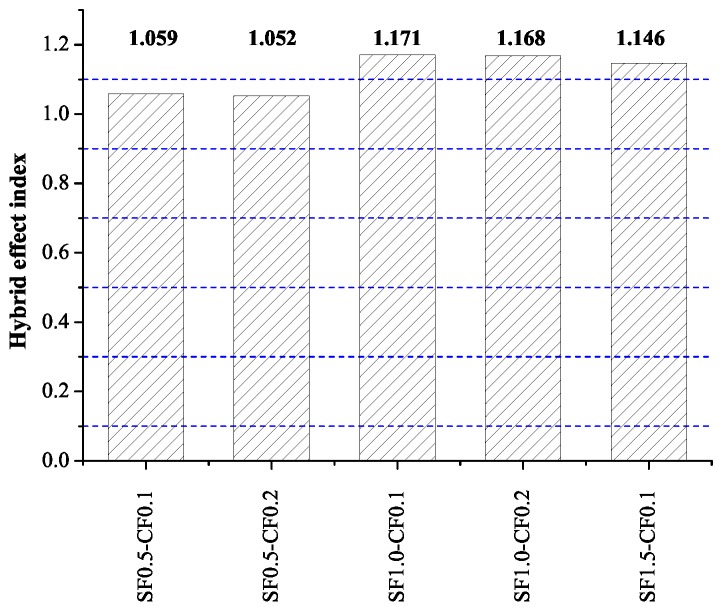
Hybrid effect index of impact toughness.

Figure 13 shows the hybrid effect index of the impact test. It can be seen that the hybrid effect index of all group of HFRC is bigger than 1,which means the hybridization of SF and CF plays a positive effect on the impact toughness of HFRC, and the hybrid effect index of SF1.0-CF0.1 is the biggest. Research of the hybridization effect of metallic and non-metallic fiber shows that steel fiber generally plays an important role in the energy absorbing mechanism (bridging action), whereas non-metallic fiber could delay the formation of the micro-cracks [13]. In this study, in terms of fiber geometry and stiffness, SF and CF belong to different dimensions and stiffness, the enhancement of the concrete performance could both be achieved in macro-crack bridging and micro-crack delaying. So positive hybrid effect exists in both the compression toughness and impact toughness.

### 4.3. Relationship between Compression Toughness and Impact Toughness

**Figure 14:**
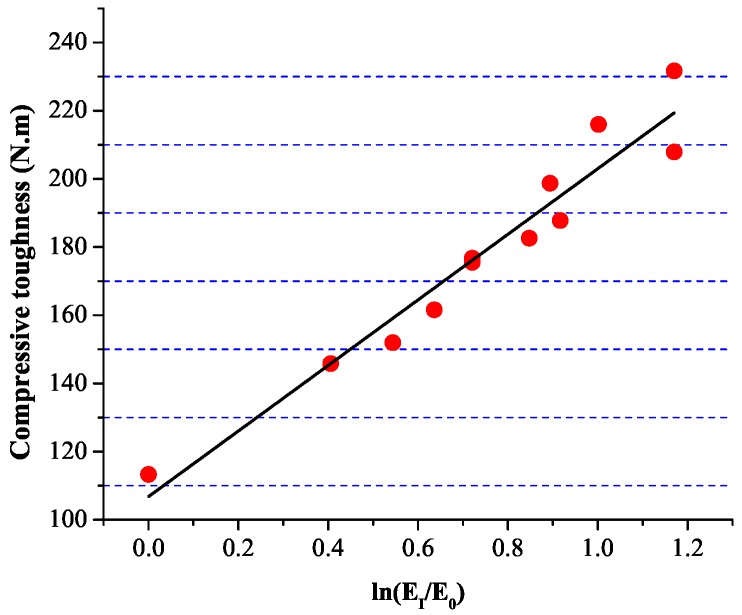
Relationship between compression toughness and impact toughness.

Relationship between the compression toughness and the impact toughness can be obtained in Figure 14. It can be observed that a logarithmic relationship between the compressive toughness ($E_{C}$) and the impact toughness ($E_{I}$) exists.

(11) $$\begin{array}{c} E_{C}=96.2ln\left(E_{I}/E_{0}\right)+106.8\;\left(R^{2}=0.935\right) \end{array}$$

where $E_{I}$ is the impact toughness of fiber reinforced concrete, $E_{0}$ is the impact toughness of natural concrete, $E_{C}$ is the compressive toughness of the fiber reinforced concrete.

## 5. Conclusions

This study evaluated the hybrid effect of steel fiber and carbon fiber on the behavior of fiber reinforced concrete. The compressive test and impact test were conducted to obtain the compressive toughness and impact toughness, and thus the hybrid effect was explored by employing the hybrid effect index. Based on the results and analysis, the following conclusions can be drawn:

- Compressive toughness of SFRC, CFRC and HFRC were evaluated, SF and CF can significantly increase the compression toughness of concrete.
- Impact toughness of SFRC, CFRC and HFRC were explored, the toughness before the first crack and the final toughness were both increased considerably. The improvement of toughness mainly lay in improving the crack resistance after the first crack.
- Analysis of the hybrid effect of steel fiber and carbon fiber in compressive toughness and impact toughness showed there was a positive hybrid effect, and SF1.0-CF0.1 showed the best performance.
- The hybridization of steel fiber and carbon fiber could enhance the concrete performance in macro-crack bridging and micro-crack delaying.
- A logarithmic relationship existed between the compressive toughness and the impact toughness.

## Figures and Tables

**Table 1:** Chemical components of the cement and fly ash.

Components	Cement	Fly Ash
$SiO_{2}$ (%)	21.38	50.15
$Al_{2}O_{3}$ (%)	5.63	30.51
$Fe_{2}O_{3}$ (%)	3.56	2.08
CaO (%)	63.72	12.5
MgO (%)	2.15	0.088
$SO_{3}$ (%)	1.75	0.4
$Na_{2}O$ (%)	1.02	1.32
LOI (%)	0.79	1.13
Total (%)	100	98.2

Where LOI is the abbreviation of Loss on Ignition.

**Table 2:** Properties of steel fiber and carbon fiber.

Fiber	Density (g/cm${}^{3}$)	Length (mm)	Elastic Modulus (GPa)	Tensile Strength (MPa)
SF	7.9	18	200	1150
CF	1.83	10	276	3000

**Table 3:** Properties of steel fiber and carbon fiber.

No.	C (kg)	FA (kg)	W (kg)	S (kg)	G (kg)	Sp (kg)	SF (kg)	CF (kg)	Slump (mm)
NC	370	50	192	767	1071	4.2	–	–	165
SF0.5	370	50	192	767	1071	4.2	40	–	153
SF1.0	370	50	192	767	1071	4.2	80	–	129
SF1.5	370	50	192	767	1071	4.2	120	–	104
CF0.1	370	50	192	767	1071	4.2	–	2	129
CF0.2	370	50	192	767	1071	4.2	–	4	115
CF0.3	370	50	192	767	1071	4.2	–	6	94
SF0.5-CF0.1	370	50	192	767	1071	4.2	40	2	118
SF0.5-CF0.2	370	50	192	767	1071	4.2	40	4	101
SF1.0-CF0.1	370	50	192	767	1071	4.2	80	2	98
SF1.0-CF0.2	370	50	192	767	1071	4.2	80	4	94
SF1.5-CF0.1	370	50	192	767	1071	4.2	120	2	86

Annotation: C—cement; FA—fly ash; W—water; S—sand; G—gravel aggregate; Sp—superplasticizer; SF—steel fiber; CF—carbon fiber; SF1.5-CF0.1 represents the concrete with 1.5% steel fiber and 0.1% carbon fiber addition.
